# Sources of persistent and mobile chemicals in municipal wastewater: a sewer perspective in Leipzig, Germany

**DOI:** 10.1007/s11356-024-33259-0

**Published:** 2024-04-18

**Authors:** Alina H. Seelig, Daniel Zahn, Thorsten Reemtsma

**Affiliations:** 1 Department of Environmental Analytical Chemistry, Helmholtz Centre for Environmental Research - UFZ, Permoserstrasse 15, 04318 Leipzig, Germany; 2 Institute of Analytical Chemistry, University of Leipzig, Linnéstrasse 3, 04103 Leipzig, Germany

**Keywords:** PMT source identification, Supercritical fluid chromatography–mass spectrometry, Organic micropollutants, Control at source, REACH chemicals, Ionic liquids, Pharmaceuticals, Dibutyl phosphate

## Abstract

Persistent and mobile (PM) chemicals spread in the water cycle and have been widely detected, yet information about their sources is still scarce. In this study, 67 PM chemicals were analyzed in 19 wastewater samples taken in the sewer system of the city of Leipzig, Germany, covering different industrial, clinical, and domestic discharges. A total of 37 of these analytes could be detected, with highly variable median concentrations between substances (median: 0.5–800 µg L^−1^) and for single substances between samples (e.g., 1,4-diazabicyclo[2.2.2]octane) by up to three orders of magnitude, with the highest single concentration exceeding 10 mg L^−1^ (*p*-cumenesulfonic acid). The emission of PM chemicals into the sewer system was classified as stemming from diffuse (14 analytes) or point sources (23 analytes), while 9 analytes fulfill both criteria. Many so-called industrial chemicals were also discharged from households (e.g., tris(2-chloroethyl) phosphate or 1H-benzotriazole). Examples for analytes showing specific sources are tetrafluoroborate (traffic-related industry and metal production and finishing), ε-caprolactam (large-scale laundry), or cyanuric acid (likely swimming pool). Furthermore, a correlation between 1-cyanoguanidine and guanylurea was observed for the traffic-related industry. This study outlines that sewer sampling can provide valuable information on the sources of PM chemicals. This knowledge is a prerequisite for their future emission control at source or substitution as an alternative to end-of-pipe treatment in municipal wastewater treatment plants.

**Graphical Abstract:**

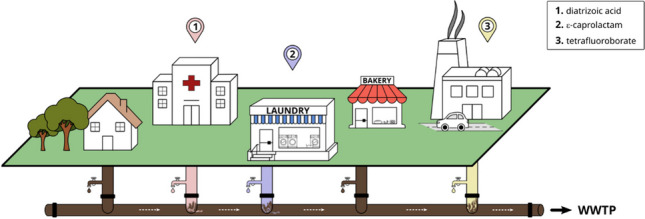

**Supplementary Information:**

The online version contains supplementary material available at 10.1007/s11356-024-33259-0.

## Introduction

By definition, persistent and mobile (PM) chemicals are poorly degraded by microbial processes and hardly sorb to natural surfaces. This is why PM chemicals can spread in the water cycle and may be found throughout the aquatic environment (Reemtsma et al. 2016). Monitoring studies demonstrated the occurrence of PM chemicals in various environmental compartments (Kolkman et al. 2021; Montes et al. 2019; Neuwald et al. 2022; Schulze et al. 2019). For example, Neuwald et al. (2022) detected 34 PM chemicals in groundwater, surface water, and bank filtrate, while Montes et al. (2019) found 23 substances in drinking water and other compartments of the water cycle. All these studies indicate the widespread presence of some PM chemicals like acesulfame (ACE) or 1-cyanoguanidine (CG) which appear in almost every sample while others were detected more sporadically but then partially in high concentrations. Since many of these chemicals are only partially removed by wastewater treatment (Neuwald et al. 2023), knowledge of their sources becomes essential to devise appropriate mitigation options like the implementation of an on-site pretreatment of the wastewater or a more focused search for replacement chemicals for specific applications if their release is found to be problematic. Furthermore, a better understanding of sources may inform future monitoring and screening campaigns by aiding in a tailored sample selection.

Screening and monitoring activities often focus on wastewater treatment plant (WWTP) effluents and larger streams. Such samples may provide an overview on chemical inventories of an entire sewer network or river catchment, thus allowing the detection of a large number of chemicals with few samples. However, they do not allow to recognize local hotspots and identify responsible emitters. Sewer samples can be used to fill this gap. In the past, these samples have been used for source identification of pharmaceuticals and personal care products (Ort et al. 2010), pathogens (Sinclair et al. 2008), or heavy metals (Jiang et al. 2022).

For industry, a distinction is made between wastewater that is discharged directly into surface water (direct discharge) and indirect discharge into municipal sewer systems and treated in municipal WWTP. Thereby, the majority of industrial wastewater is discharged indirectly. While constituents of such industrial discharges may be detected in WWTP effluents, their respective industrial origin remains hidden.

In Germany, wastewater companies regularly control indirect industrial discharges into their sewer system through “qualified random samples” which are composites of five samples taken over a period of up to 2 h (AbwV, 2004). These samples are used to control compliance of the discharger with the respective discharge permits and are usually based on sum parameters (e.g., pH value, COD, BOD). Single chemicals or classes of organic contaminants are not routinely analyzed. However, sewer samplings and the samples taken for discharge control from indirect dischargers may offer the potential for identifying sources of specific chemicals. This would be of particular importance for compounds that are insufficiently removed in WWTP and affect the quality of the water cycle.

This study explores if samples taken to control indirect discharges are useful for identifying sources of PM chemicals in municipal wastewater. The generated data is used to elucidate whether the PM chemicals analyzed (a) originate from diffuse or point sources and (b) what kind of point source a chemical originates from. By doing so, the potential of sewer samples for source tracking is investigated, and its potential to improve monitoring activities and chemicals management is discussed.

## Materials and methods

### Chemicals and PM standards

Methanol (MeOH) and ammonium formate were UPLC-MS grade from Biosolve (Valkenswaard, Netherlands). Also, formic acid (99%) was purchased from Biosolve (Valkenswaard, Netherlands). Ammonium hydroxide (LC–MS grade) was obtained from Honeywell (Charlotte, NC, USA). Ultrapure water from a Milli-Q system (Merck KGaA, Darmstadt, Germany) was used. All stock standards with a concentration of 1 mg mL^−1^ were prepared in methanol:water (50:50, *v:v*) or methanol (depending on solubility) stored at − 20 °C. A list of all 67 PM standards with suppliers, purities, and physicochemical properties is shown in Table S1 in the SI. For the estimation of physicochemical properties (logD values), ACD/Percepta (ver. 2020.1.2) was used.

### Samples and PM chemicals

Nineteen wastewater samples were collected in the sewer system of Leipzig, Germany, from different catchment areas categorized in clinical, domestic, and industrial wastewater. The samples were collected from indirect discharger in combined and separated sewers. Samples categorized as traffic-related industry can include samples from gas stations, car washes, motor vehicle workshop, transportation companies, etc. They were sampled in two different time periods in March and October 2022. All samples were taken in 500-mL narrow neck round shoulder amber glass bottles (Th. Geyer, Renningen, Germany), filled to the top and stored at 4 °C. Replicates for each sample site have not been taken.

PM chemicals were categorized as follows: Those listed by ECHA with fields of application were categorized as “industrial chemicals” (European Chemicals Agency (ECHA) 2023a). The group of “pharmaceuticals” was categorized on the basis of the documentation of the analytes in the “Model List of Essential Medicines” of the WHO or in DrugBank (Wishart et al. 2018; World Health Organization (WHO) n.d.). Substances that fulfilled both criteria were listed in the group “both.” Analytes that were not listed by either ECHA or WHO/DrugBank or those listed by ECHA without applications were listed in a separate group (“not specified”). All literature data used for categorization for each single analyte including also some articles from peer-reviewed journals are listed in Table S2.

### Sample preparation

From sampling till delivery samples were stored at 4 °C. Afterwards, the samples were centrifuged two times. The first step was for 10 min (5000 rpm, rt) and afterwards for another 10 min (13,000 rpm, 4 °C). The samples were then diluted in several steps in methanol:Milli-Q water (50:50) and spiked with reference standards for quantification (Table S3). Each sample was measured ones and stored at -20 °C afterwards.

### Analytical method

Sample analysis was performed by using a supercritical fluid chromatography (SFC, Acquity UPC^2^ system) coupled to a tandem mass spectrometry (TQXS) both from Waters (Milford, MA, USA). The analytical method was based on Schulze et al. (2020). The compounds were separated using a Waters Acquity UPC^2^ BEH (3.0 × 100 mm, 1.7 µm, 130 Å) or Waters Torus Diol column (3.0 × 150 mm, 1.7 µm, 130 Å) which were operated at 55 °C. Table S4 lists the compounds of interest analyzed with which column. Both methods run with an eluent flow rate of 1.5 mL min^−1^ and a make-up flow rate of 0.3 mL min^−1^. The gradients for both methods are listed in Table S5. The method using the Waters Acquity UPC^2^ BEH column runs for 17.2 min and with a mobile phase of eluent A (CO_2_) and eluent B (95% methanol, 5% Milli-Q water, 10 mM ammonium formate). The second method (Waters Torus Diol column) runs for 9 min and with a mobile phase of eluent A (CO_2_) and eluent B (90% methanol, 10% Milli-Q water, 0.05% of a 25% ammonium hydroxide solution).

### Data analysis

Data analysis was performed using the software TargetLynx from Waters (Milford, MA, USA). For quantification, a 7-point external calibration was used including apparent recoveries by spiking the samples with reference standards in different concentrations additionally (Table S3). Calibration points with a deviation > 25% were excluded. Isotope-labeled standards are rarely available for PM chemicals and the use of isotope-labeled standards of other chemicals with similar retention times is not feasible for PM chemicals (Müller et al. 2020). Validation data are shown in Table S6.

## Results and discussion

### PM chemicals in the sewer system

In this study, 67 suspected or known PM compounds were quantified by SFC-MS/MS in 19 wastewater samples taken in the sewer system of the city of Leipzig, Germany. The list of analytes comprises industrial chemicals, pharmaceuticals, and substances used in several fields, including some transformation products (TPs). The selection of PM chemicals was made on the basis of a previous screening study (Neuwald et al. 2021).

**Fig. 1:**
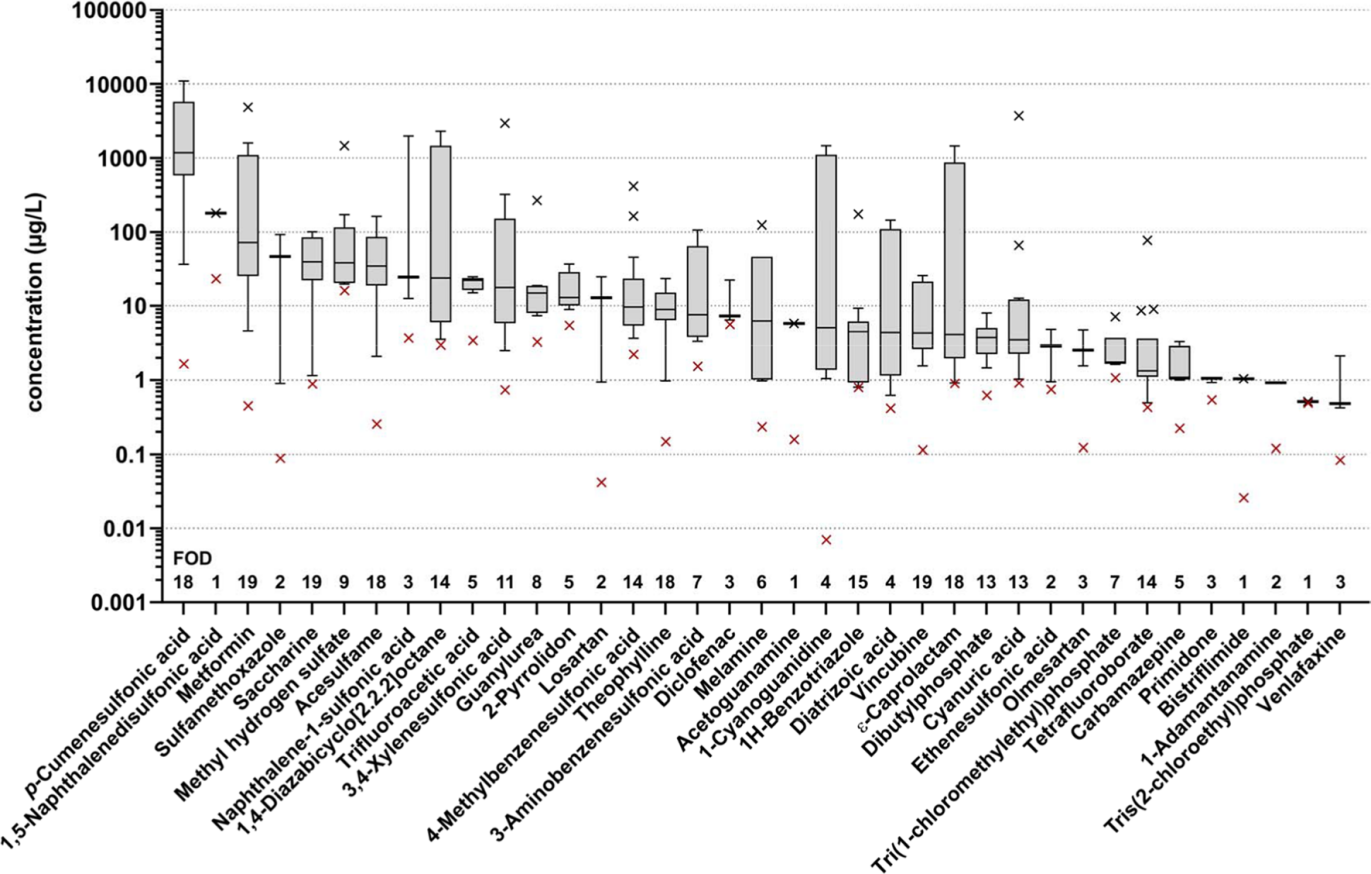
Concentrations of PM chemicals in the 19 sewer samples taken in March and October 2022. The gray crosses are representing outliers and the red crosses the limit of detection (LOD). FOD, frequency of detection. Whiskers were calculated with Tukey method

In total, 37 analytes (54%) were detected in at least one of the wastewater sample (Fig. 1). The detected substances have a logD value (pH 7.5) between -5.4 (1,5-naphthalenedisulfonic acid (NDSA)) and 2.3 (tri(1-chloromethylethyl) phosphate (TCPP)). Concentrations of PM chemicals in sewer systems varied widely, with maximum concentrations ranging from 0.5 µg L^−1^ for tris(2-chloroethyl) phosphate (TCEP) to 11 mg L^−1^ for *p*-cumenesulfonic acid (CSA). Additionally, concentrations of single analytes also differed widely between samples. Highest variability is visible for the industrial chemical CSA from 36 µg L^−1^ to 11 mg L^−1^ and the pharmaceutical metformin (MET) from 5 µg L^−1^ to 5 mg L^−1^. Furthermore, three chemicals (CG, 1,4-diazabicyclo[2.2.2]octane (DABCO), and ε-caprolactam (εCL)) showed an interquartile range that spans over three orders of magnitude.

### Comparison between industrial and domestic wastewater

The sewer samples analyzed were highly variable in both, composition and concentration: 22 of the 37 detected compounds analyzed (49%) were detected in less than 50% of the samples, while 15 compounds (41%) spanned up to three orders of magnitude in concentration. This raises the question if this inhomogeneity is connected to a certain sample type investigated herein: clinical, domestic, or industrial samples. It was reported previously that domestic and clinical wastewater often shows a similar pattern of organic micropollutants (Carraro et al. 2016). Therefore, the domestic and clinical samples were combined in one group in this study (Fig. S1) and compared with the samples of industrial discharges.

**Fig. 2:**
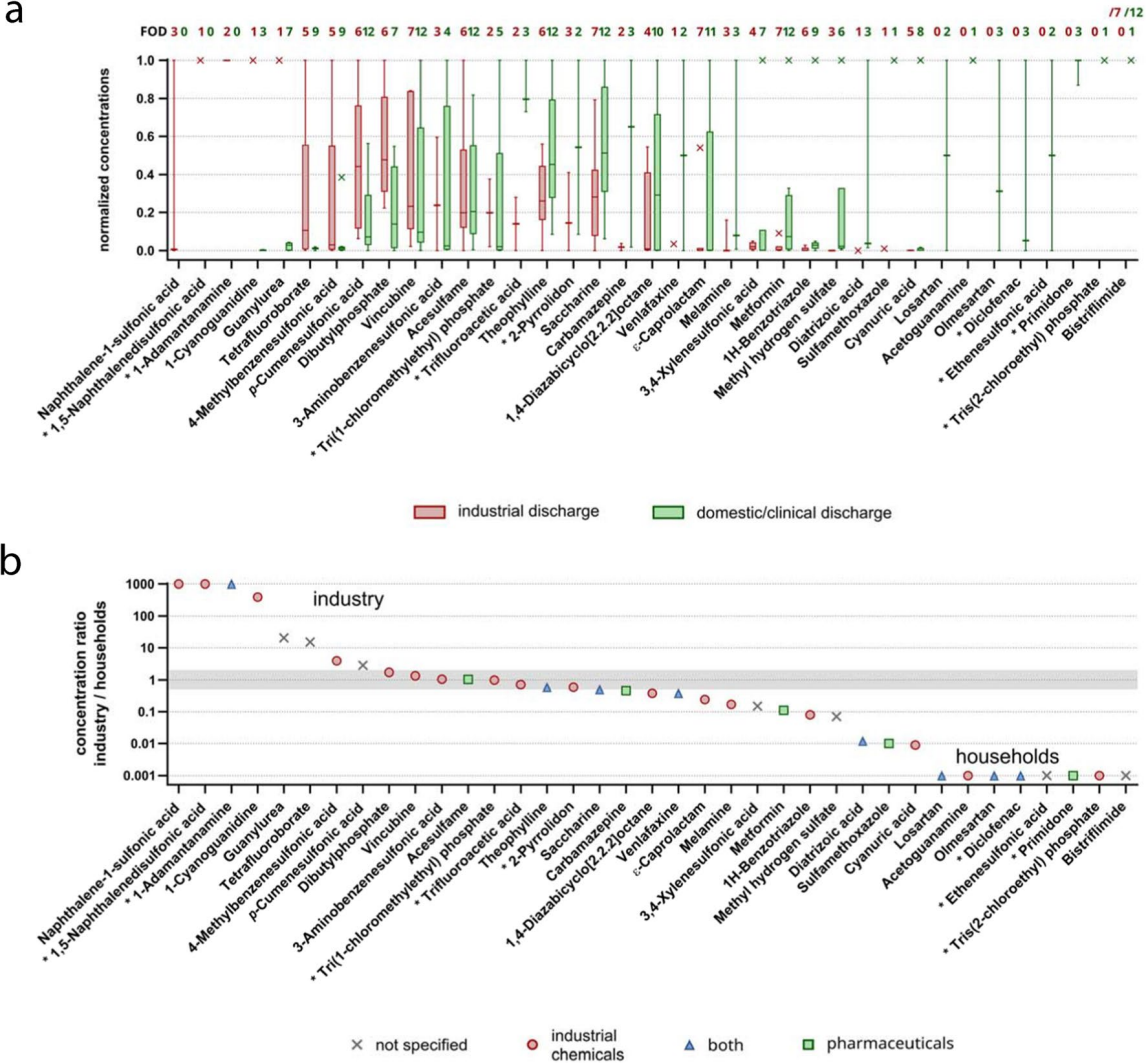
Comparison between industrial and domestic/clinical wastewater. **a** Normalized concentrations of PM chemicals in industrial (red) and domestic/clinical (green) wastewater. Normalization was carried out from the lowest to the highest detected concentration per analyte. Outliers are illustrated by crosses. Frequency of detection (FOD) given above the columns. Whiskers were performed with Tukey method. **b** Ratio between the mean concentration in industrial and domestic wastewater samples. Analytes that were only detected in industrial samples were set to a value of 1000, and those ones only in domestic/clinical wastewater samples were set to a value of 0.001. Intermediate range, 0.5–2

In Fig. 2a the normalized concentrations for both domestic/clinical and industrial wastewater samples are illustrated. Concentrations of each analyte were normalized to its highest concentration in the sample set. Analytes on the left side of Fig. 2a were only found in the industrial samples, while those on the right were found only in the domestic/clinical discharge. Thereby, the concentrations in industrial samples are decreasing from left to right and increasing for domestic/clinical samples. One example is CSA found in almost all samples (6/7 industrial samples, 12/12 domestic/clinical samples), but the median concentration in industrial samples (2.4 mg L^−1^) is four times higher than in domestic samples (0.6 mg L^−1^). Compounds in the middle of Fig. 2a are equally present in industrial and in domestic/clinical wastewater.

To assess if emission patterns correspond to documented applications, the chemicals analyzed were categorized into four groups (not specified, industrial chemicals, pharmaceuticals, and both) based on literature data (Table S2).

In Fig. 2b, the analytes are sorted by the ratio between the mean concentration of industrial to domestic/clinical samples. Thereby, PM chemicals which occur preferentially in domestic/clinical wastewater have a ratio of < 1 and the ones which are mainly found in industrial wastewater have a ratio of > 1. However, the calculated ratios were found to poorly correspond to the assigned use categories of the chemicals. This may have several reasons: (a) Literature information on chemical uses is often very general and lacks specificity or is largely missing. (b) Chemicals used for the manufacture of consumables may be released not only during their synthesis or the manufacture of that consumable but also during the life cycle of that consumable (e.g., acetoguanamine or TCEP). (c) About 22% of the study compounds turned out to be used both as pharmaceutical and as industrial chemical.

Analyzing samples from sewer systems and discharge control may, thus, complement literature information on chemicals use.

### Distribution of PM chemicals in different catchment areas

**Fig. 3:**
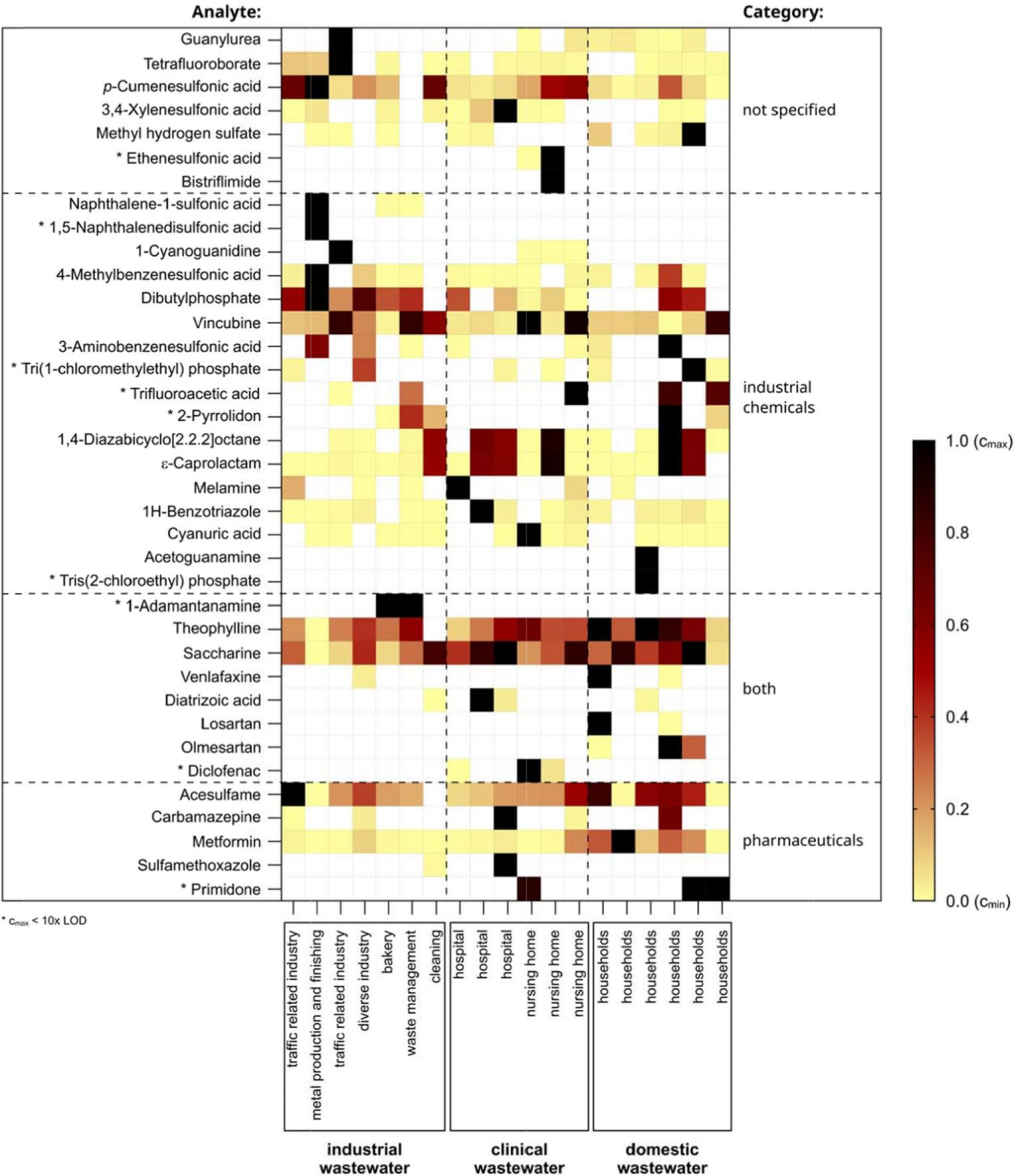
Heatmap of normalized concentrations for the 37 PM chemicals found in the 19 sewer samples. Normalization was performed from the lowest to the highest detected concentration for every analyte separately. White, no detection; concentration < LOD. *Analytes with a maximum concentration < 10 × LOD

Since the detection of many PM chemicals does not seem to correspond well with the literature information on their uses, a closer look into high concentration samples may help to identify specific sources of individual PM chemicals (Fig. 3). Normalization was performed between the lowest (0) and highest (1) concentration for each analyte in the 19 samples.

Figure 3 shows that analytes may have (a) diffuse sources and are, thus, detected in at least 50% of the samples, or (b) point source with high concentration (> 10 times the median) in specific industrial or commercial wastewater samples (Table 1), or (c) have diffuse as well as point sources.

**Table 1:** Classification of analytes as diffuse and/or point source. Diffuse source: detection in at least 50% of the samples. Point source: maximum concentration must be 10 times greater as the median. Non-detects were set to LOD. The potential origins of point sources are derived from the data shown in Fig. 3 (highest detected concentration)

Analyte	Diffuse source^a^/(*c*_median_ in µg L^−1^)	Point source^b^/(*c*_max_ in µg L^−1^)	Main emitter (*c*_max_) in this sample set
Tetrafluoroborate	Yes (1.3)	Yes (77)	Traffic-related industry
Bistriflimide		Yes (1.0)	Nursing home
Dibutyl phosphate	Yes (3.7)		
ε-Caprolactam	Yes (4.1)	Yes (1400)	Large-scale laundry
1-Cyanoguanidine		Yes (1500)	Traffic-related industry
Guanylurea		Yes (300)	Traffic-related industry
Acetoguanamine		Yes (5.8)	Households
Melamine		Yes (120)	Hospitals
Cyanuric acid	Yes (3.5)	Yes (3700)	Nursing home
1,4-Diazabicyclo[2.2.2]octane	Yes (24)	Yes (2300)	Nursing home
Methyl hydrogen sulfate		Yes (1500)	Households
1H-Benzotriazole	Yes (4.5)	Yes (170)	Hospitals
Vincubine	Yes (4.3)		
Diatrizoic acid		Yes (140)	Hospitals
*p*-Cumenesulfonic acid	Yes (1200)	Yes (11,000)	Metal production and finishing
4-Methylbenzenesulfonic acid	Yes (10)	Yes (420)	Metal production and finishing
3,4-Xylenesulfonic acid	Yes (18)	Yes (3000)	Hospitals
Naphthalene-1-sulfonic acid		Yes (2000)	Metal production and finishing
3-Aminobenzenesulfonic acid		Yes (110)	Households
Metformin	Yes (72)	Yes (4900)	Households
Theophylline	Yes (9.0)		
Losartan		Yes (25)	Households
Olmesartan		Yes (4.7)	Households
Venlafaxine		Yes (2.1)	Households
Carbamazepine		Yes (3.3)	Households
Sulfamethoxazole		Yes (93)	Hospitals
Saccharine	Yes (39)		
Acesulfame	Yes (35)		

^a^FOD > 50%

^b^*c*_max_ > 10 × *c*_median_

According to this definition, 14 of the 37 detected substances (38%) fall into group (a) with diffuse sources. Examples are MET, theophylline (THEO), saccharine (SAC), or ACE occurring in wide concentration ranges (up to three orders of magnitude; MET and SAC, 19/19; THEO and ACE, 18/19). While MET and ACE only classified as pharmaceuticals, THEO and SAC are used both industrially and pharmaceutically and listed by ECHA and in medical databases (e.g., model list of essential medicines by WHO or DrugBank), which explains the large frequency of detection. Nevertheless, it should be mentioned that ACE and SAC are sweeteners regardless of their classification in this study and are largely used as such. Other compounds with diffuse sources are DABCO (14/19) and vincubine (VIN, 19/19) with median concentrations of 24 (DABCO) and 4 µg L^−1^ (VIN). Both are categorized here as industrial compounds and used in multiple areas and products. DABCO is used, for example, in the plastic production as adhesives, in food packaging or coating products and may leach from such materials during their use. Furthermore, DABCO is used for building and construction work, for the manufacture of machinery and vehicles and as pH regulators or adsorbents (European Chemicals Agency (ECHA) 2023b). Information on the use of VIN (2,2,6,6-tetramethyl-4-piperidinone), a natural metabolite in plants (Li et al. 2011), are limited; it is used as an intermediate for the production of other chemicals (European Chemicals Agency (ECHA) 2023c).

For some PM chemicals, the data collected in this study (Fig. 3) suggest that they are primarily used in specific industries or applications with a high discharge concentration. These compounds are discussed in more detail below:

#### Tetrafluoroborate

Tetrafluoroborate (BF_4_) is an anion commonly used in ionic liquids (ILs) (Freire et al. 2010). Due to their various combinations of cations and anions, ILs can have tunable properties and are used in a multitude of processes. In the sewer samples, BF_4_ was widely detected in concentrations around 1 µg L^−1^ indicating diffuse emission and widespread use. Two samples connected to the traffic related industry (e.g., gas stations, car washes, motor vehicle workshop, or transportation companies) as well as a metal production and finishing related sample, however, showed significantly higher concentrations of 8.6, 9.0, and 77 µg L^−1^ (Fig. S2).

#### ε-Caprolactam

εCL is mainly used as the monomer for polycaprolactam production (polyamide 6, PA6), which is used for textile fibers, among others (Turk et al. 2016) and is regulated under REACH (European Chemicals Agency (ECHA) 2023d). Here, εCL could be detected in every sample. Furthermore, εCL was found with significantly elevated concentrations in six effluents (cleaning (782 µg L^−1^), two hospitals (861 µg L^−1^, 900 µg L^−1^), nursing home (1342 µg L^−1^), and two households (905 µg L^−1^, 1447 µg L^−1^)) (Fig. S2).

PA6 was reported to contain 0.1–10% with the monomer εCL (Zilio-Grandi et al. (1969)). Considering the large amounts of polyamide produced worldwide each year (5.9 million tons in 2021 (Statista 2023)), this may lead to a regular release of εCL during washing processes.

#### 1-Cyanoguanidine and guanylurea

CG, which was detected in four of 19 samples, has a wide range of application fields, e.g., fertilizer production, textile industry, and dyes (Schulze et al. 2019). In the field of dyes, it is also used for water-based coagulation to remove coating residues in the automotive industry (Molz et al. 1996). This agrees to the fact that CG was primarily found in discharges of the traffic related industry (Fig. 3, Table 1).

The maximum concentration of guanylurea (GUA) was found in the same sample as the maximum of CG, suggesting that it occurred here as an impurity, as CG is produced from GUA (Klapötke and Miró Sabaté 2010). Besides, GUA is well known as the major microbial transformation product (in WWTPs under anaerobic conditions) of the pharmaceutical MET (Trautwein and Kümmerer 2011). This may explain why MET was detected in five of six household wastewater samples (median concentration of 15 µg L^−1^) in which MET was found at highest concentration (median concentration of 1500 µg L^−1^, Fig. 3).

#### Cyanuric acid

Cyanuric acid (CA) is primarily used as chlorine stabilizer for disinfection in swimming pools (Braekevelt et al. 2011) and known as a major transformation product of melamine (MEL) (Zhu and Kannan 2020). However, concentrations of CA in the 19 sewer samples were not correlated to MEL concentrations. Here, CA had diffusive as well as point sources (Table 1). While most samples had similar concentrations (median concentration of 4 µg L^−1^), one sample from a nursing home showed an exceptionally high concentration of 3700 µg L^−1^. Possibly, this nursing home includes an in-house swimming pool, causing the CA discharge.

#### Dibutyl phosphate

Dibutyl phosphate (DBP) is the main decomposition product of tributyl phosphate (TBP) but also itself used as plasticizer (Quintana et al. 2006). DBP and TBP are regulated under REACH. ECHA lists a range of uses in long-life materials (e.g., furniture or toys), in cardboard and plastic products or electronic equipment (European Chemicals Agency (ECHA) 2023e). The wide use of DBP (and TBP) is well reflected in the occurrence of DBP in 13 of the 19 sewer samples of this study (Fig. 3). Further on, it exhibits a limited variability in concentrations (4 µg L^−1^ ± 2 µg L^−1^) in 47% of the samples and is found in almost every industrial sample.

#### *p*-Cumenesulfonic acid

CSA (4-isopropylbenzenesulfonic acid) showed the highest concentration in a single sample (11 mg L^−1^). It was detected in almost every sample, but with highly variable concentrations ranging over two orders of magnitude. This is also demonstrated by the chemicals vast range of applications. CSA is used widely in industry, in manufacturing, and in household products like bleach and air freshener or is applied for the production of surfactants (European Chemicals Agency (ECHA) 2022; US Environmental Protection Agency (EPA) n.d.).

In this study, highest concentrations of CSA were found in the discharge of nursing homes and samples belonging to the traffic-related and cleaning industry.

### Potential of sewer samples for source identification

This study outlines the potential of sewer samples for the identification of sources of chemicals in municipal wastewater. The main advantage of sewer samples is the clear and direct connection of positive detects to potential sources, with often only one trade or industry connected to the sampling point. The high concentrations found in proximity of a point source also make sample enrichment superfluous. This reduces the workload in the laboratory and eliminates the risk of losing analytes which are difficult to enrich, such as many PM chemicals (Zahn et al. 2020).

The main limitation of such a sewer monitoring is due to the grab sampling or sampling over only a limited period of 2 h or less. By such a sampling, chemicals from event-driven or otherwise discontinuous discharges may easily be overlooked. Taking repeated samples from the same site, ideally by automated generation of composite samples, would avoid this weakness, but requires much larger effort and costs. It appears promising to extend the monitoring for PM chemicals to samples taken by wastewater utilities for the control of indirect dischargers. Much could be learned with respect to the source of chemicals found in municipal wastewater if these potentially large numbers of samples would be used more intensively.

## Conclusions

The sewer-based monitoring approach for 67 PM chemicals, although limited to 19 samples, provided information on specific sources for seven of the investigated PM chemicals and showed the widespread use of 14 of the studied substances at the same time. The detected sources were compared with the literature information on their applications. For so-called industrial chemicals, it is not necessarily the site of production or industrial use that acts as a point of emission into the sewer system; rather, the use of certain products containing that chemical can lead to a more diffuse emission of industrial chemicals, e.g., from households.

Specific sources could be identified for 23 PM chemicals (e.g., BF_4_ (traffic-related industry or metal production and finishing) or CG (traffic-related industry)), while 14 showed a diffusive pattern (e.g., THEO or VIN). Of these, nine analytes exhibited both, sources of elevated concentration and a broad diffusive emission. Information gathered from sewer analysis or the analysis of samples taken during the control of indirect dischargers should improve our knowledge of the major points of discharge. This can be instrumental for a future control of problematic PM chemicals, either by improved on-site treatment or by chemical substitution.

## Supplementary Information

Below is the link to the electronic supplementary material.

Supplementary file1 (PDF 651 KB)

## Data Availability

Data are made availabule upon request.
