# Supplementary material for: Sources of persistent and mobile chemicals in municipal wastewater: a sewer perspective in Leipzig, Germany
Source: Environ Sci Pollut Res Int. 2024 Apr 18;33(22):11009–17. doi: 10.1007/s11356-024-33259-0 (PMC13415350; doi:10.1007/s11356-024-33259-0)
Supplement: Supplementary file 1 — Supplementary file1 (PDF 651 KB) [file 11356_2024_33259_MOESM1_ESM.pdf]

Supplementary Information

Sources of persistent and mobile chemicals in municipal wastewater: a sewer perspective in Leipzig, Germany

Environmental Science and Pollution Research

A. H. Seelig[1], D. Zahn[1], T. Reemtsma[1,2]

[1] Helmholtz Centre for Environmental Research - UFZ, Department of Analytical Chemistry, Permoserstrasse 15, 04318 Leipzig, Germany

[2] Institute of Analytical Chemistry, University of Leipzig, Linnéstrasse 3, 04103 Leipzig, Germany

* Corresponding author: Thorsten Reemtsma, thorsten.reemtsma@ufz.de

Table of contents

Material and Methods

Table S1: List of analyzed PM chemicals with substance information. S2

Table S2: Categorization and used literature of detected analytes. S4

Table S3: Dilution and spike concentrations per sample. S7

Table S4: SFC-MS parameter of analyzed substances. S8

Table S5: Eluent gradient of both used methods. S9

Method validation S9

Results and Discussion

Table S6: Method validation parameters. S10

Fig. S1: Concentrations of PM chemicals in domestic (green) and clinical (blue) discharge. S12

Fig. S2: Concentrations of selected analytes. S13

Table S1 List of analyzed PM chemicals with substance information.

Name	Abbreviation	CAS	Supplier	Purity in %	Detected?	logD (pH 7.5)[a)]
acesulfame	ACE	33665-90-6	Sigma-Aldrich	> 99	yes	-2.77
acetoguanamine	AG	542-02-9	Sigma-Aldrich	98	yes	-0.18
1-adamantanamine	ATA	768-94-5	fluorochem	97	yes	-0.33
3-aminobenzenesulfonic acid	MA	121-47-1	Dr. Ehrenstorfer	99	yes	-4.29
1H-benzotriazole	BTZ	95-14-7	Dr. Ehrenstorfer	99.5	yes	1.43
bistriflimide	NtF2	82113-65-3	abcr	97	yes	0.51
carbamazepine	CBZ	298-46-4	Sigma-Aldrich	≥ 98	yes	2.28
1-cyanoguanidine	CG	461-58-5	Acros	99.9	yes	-1.03
cyanuric acid	CA	108-80-5	Sigma-Aldrich	> 98	yes	-3.46
diatrizoic acid	DZA	117-96-4	Sigma-Aldrich	≥ 98	yes	-1.01
1,4-diazabicyclo[2.2.2]octane	DABCO	280-57-9	Sigma-Aldrich	> 99	yes	-1.20
dibutylphosphate	DBP	107-66-4	J&K	97	yes	-1.99
diclofenac	DCF	15307-86-5	Sigma-Aldrich	-	yes	1.29
ε-caprolactam	εCL	105-60-2	Sigma-Aldrich	99	yes	-0.02
ethenesulfonic acid	ESA	1184-84-5	abcr	25% in H2O	yes	-4.56
guanylurea	GUA	141-83-3	Acros Organics	98	yes	-1.81
losartan	LOS	114798-26-4	Ambeed	98	yes	1.56
melamine	MEL	108-78-1	Sigma-Aldrich	> 99	yes	-1.18
metformin	MET	657-24-9	MP	≥98	yes	-3.33
4-methylbenzenesulfonic acid	PTSS	104-15-4	MP	≥ 99	yes	-3.35
methyl hydrogen sulfate	MHS	75-93-4	Sigma-Aldrich	> 99	yes	-4.87
1,5-naphthalenedisulfonic acid	NDSA	81-04-9	abcr	98	yes	-5.38
naphthalene-1-sulfonic acid	NSA	85-47-2	fluorochem	-	yes	-2.69
olmesartan	OLM	144689-24-7	Sigma-Aldrich	> 98	yes	-0.28
p-cumenesulfonic acid	CSA	16066-35-6	BLDpharm	98	yes	-2.79
primidone	PRI	125-33-7	Fluka	-	yes	0.61
2-pyrrolidone	2PYR	616-45-5	Roth	> 99.5	yes	-0.80
saccharine	SAC	81-07-2	Sigma-Aldrich	99.9	yes	-1.30
sulfamethoxazole	SMX	723-46-6	Fluka	-	yes	-0.63
tetrafluoroborate	BF_4	14874-70-5	abcr	95	yes	-
theophylline	THEO	58-55-9	Sigma-Aldrich	-	yes	0.10
trifluoroacetic acid	TFA	76-05-1	Biosolve	-	yes	-3.09
tris(2-chloroethyl) phosphate	TCEP	115-96-8	fluorochem	97	yes	1.42

tris(1-chloromethylethyl) phosphate	TCPP	13674-84-5	Sigma-Aldrich	-	yes	2.31
venlafaxine	VEN	93413-69-5	Sigma-Aldrich	≥ 98	yes	1.53
vincubine	VIN	826-36-8	J&K	99	yes	-0.55
3,4-xylenesulfonic acid	XSA	1300-72-7	Sigma-Aldrich	> 91	yes	-3.03
2-acrylamido-2-methylpropanesulfonic acid	AAMPS	15214-89-8	Sigma-Aldrich	99	no	-5.40
3-(acryloyloxy)-1-propanesulfonic acid	AOPSO3	31098-20-1	fluorochem	99	no	-4.74
3-(allyloxy)-2-hydroxy-1-propanesulfonic acid	AHP	52556-42-0	Sigma-Aldrich	39-42<br>40% wt in H2O	no	-5.29
ametryn	ATY	834-12-8	Sigma-Aldrich	98.5	no	3.04
4-amino-3-methylbenzenesulfonic acid	AMB	63450-43-1	TCI	> 98	no	-3.80
3,5-bis(methoxycarbonyl)benzenesulfonic acid	SIP	138-25-0	Alfa Aesar	98	no	-3.02
climbazole	CLIM	38083-17-9	HPC	99.8	no	3.32
dicyclohexyl sulfosuccinate	DCHSS	137361-04-7	Sigma-Aldrich	> 98	no	-1.74
2-[2-(dimethylamino)ethoxy]ethanol	DMAEE	1704-62-7	Sigma-Aldrich	98	no	-1.96
1,3-di-o-tolylguanidine	DIOTOG	97-39-2	Sigma-Aldrich	99	no	2.33
1,3-diphenylguanidine	DPG	102-06-7	Merck	99.2	no	1.62
3,5-ditertbutyl salicylic acid	DTBSA	19715-19-6	Sigma-Aldrich	97	no	2.14
1-ethylpyridinium	PYC2	15302-96-2	fluorochem	99	no	-3.24
ethyltrimethylammonia	AC5	15302-88-2	trc	-	no	-3.10
gabapentin lactam	GALA	64744-50-9	fluorochem	98	no	1.40
hexafluorophosphate	PF6	16919-18-9	Sigma-Aldrich	> 98,5	no	-
2-[4-(2-hydroxyethyl)-1-piperazine]ethanesufonic acid	HEPES	7365-45-9	Sigma-Aldrich	> 99.5	no	-5.59
3-(methacryloylamino)-N,N,N-trimethyl-1-propanaminium chloride	MAPTAC	51410-72-1	abcr	50 aq	no	-3.73
4,4'-methylenedianiline	MDA	101-77-9	Sigma-Aldrich	≥97	no	1.68
1-methyl-1-octylpyrrolidinium	PLC9	927021-43-0	Combi-Blocks	95	no	-0.14
2-methyl-2-propene-1-sulfonic acid	MPSA	1561-92-8	Sigma-Aldrich	98	no	-4.38
N-[3-(dimethylamino)propyl]-2-methylacrylamide	MAPMA	5205-93-6	fluorochem	95	no	-1.13
N,N-dibutyl-N-methyl-1-butanaminium	AC13	3085-79-8	Sigma-Aldrich	70-80<br>75 wt % in H2O	no	-1.25
N,N,N-trimethyl(phenyl)methaneaminium	BETMAC	14800-24-9	Sigma-Aldrich	97	no	-2.32
oxipurinol	OXI	2465-59-0	Sigma-Aldrich	> 98	no	-0.39
perfluorobutanoic acid	PFBA	375-22-4	Wellington	-	no	-1.13
perfluoropropionic acid	PFPrA	422-64-0	Wellington	98	no	-2.24
1-propylpyridinium	PYC3	45705-28-0	trc	-	no	-3.01
tricyanomethanide	TCM	36603-80-2	trc	97.5	no	0.02
tris(trifluoromethylsulfonyl)methanide	TFSM	114395-69-6	TCI	> 98	no	7.27

[a] calculation with ACD/Percepta (ver. 2020.1.2)

Table S2 Categorization and used literature of detected analytes.

Analyte	Category	Uses based on literature	Literature
acesulfame	pharmaceuticals	- artificial sweetener	Schulze et al. (2019), Wishart et al. (2018)
acetoguanamine	industrial chemicals	- at industrial sites and in manufacturing<br>- in manufacturing of melamine resins<br>- stabilizer for formaldehyde solutions<br>- in the decorative layer of high-pressure laminates	ECHA (2023), Schulze et al. (2019)
1-adamantanamine	both	- antiparkinson agent<br>- at industrial sites and in manufacturing	ECHA (2023), Wishart et al. (2018)
3-aminobenzenesulfonic acid	industrial chemicals	- in formulation or re-packing and at industrial sites<br>- for the synthesis of copolymers	Amer et al. (2016), ECHA (2023)
1H-benzotriazole	industrial chemicals	- anticorrosive agents	Davis et al. (1977), ECHA (2023)
bistriflimide	not specified	- in ionic liquids	Cornet et al. (2009)
carbamazepine	pharmaceuticals	- against epilepsy or seizures<br>- to tread bipolar or related disorders	WHO (n.d.), Wishart et al. (2018)
1-cyanoguanidine	industrial chemicals	- in articles, by professional workers, in formulation or re-packing, at industrial sites and in manufacturing<br>- fertilizer production, textile industry, and dyes	ECHA (2023), Molz et al. (1993), Schulze et al. (2019)
cyanuric acid	industrial chemicals	- by consumers, in articles, by professional workers, in formulation or re-packing, at industrial sites and in manufacturing<br>- as chlorine stabilizer for disinfection	Braekevelt et al. (2011), ECHA (2023)
diatrizoic acid	both	- contrast agent<br>- professional workers, in formulation or re-packing, at industrial sites, and in manufacturing	Chellquist et al. (1997), ECHA (2023), Wishart et al. (2018)
1,4-diazabicyclo[2.2.2]octane	industrial chemicals	- by consumers, in articles, by professional workers, in formulation or re-packing, at industrial sites, in manufacturing<br>- as catalyst in organic synthesis<br>- process regulator, in vulcanization or polymerization processes	Basavaiah et al. (1996), Baylis and Hillman (1972), ECHA (2023), Schulze et al. (2019)
dibutylphosphate	industrial chemicals	- in articles, by professional workers (widespread uses), in formulation or re-packing, at industrial sites and in manufacturing<br>- in ionic liquids	ECHA (2023), van Soestbergen et al. (2014), Zheng et al. (2020)
diclofenac	both	- in the treatment of pain and inflammation	ECHA (2023), Wishart et al. (2018)

ε-caprolactam	industrial chemicals	- for polycaprolactam production<br>- by consumers, in articles, by professional workers, in formulation or re-packing, at industrial sites and in manufacturing	ECHA (2023), Turk et al. (2016)
ethenesulfonic acid	not specified	- for synthesis of poly(vinyl sulfonic acid)	Sepehrianazar and Güven (2023)
guanylurea	not specified	- in medical applications and in several industrial fields<br>- for the synthesis of flame retardants	Kasetti and Bharatam (2013), Oberley (1983)
losartan	both	- antihypertensive medicines<br>- medicines used in heart failure<br>- to treat diabetic nephropathy	ECHA (2023), WHO (n.d.), Wishart et al. (2018)
melamine	industrial chemicals	- in articles, by professional workers, in formulation or re-packing, at industrial sites, and in manufacturing<br>- in dinnerware, kitchenware, adhesives, and laminates<br>- in flame retardant	Braekevelt et al. (2011), ECHA (2023), Zhu and Kannan (2020)
metformin	pharmaceuticals	- against type 2 diabetes mellitus<br>- for insulin resistance in polycystic ovary syndrome (PCOS)	WHO (n.d.), Wishart et al. (2018)
4-methylbenzenesulfonic acid	industrial chemicals	- by professional workers, in formulation or re-packing, at industrial sites, and in manufacturing<br>- in cleaning and personal care products	ECHA (2023), Stanton et al. (2007)
methyl hydrogen sulfate	not specified	- in ionic liquids	Fang et al. (2022)
naphthalene-1-sulfonic acid / 1,5-naphthalenedisulfonic acid	industrial chemicals	- in formulation or re-packing and at industrial sites (NDSA)<br>- in the synthesis of azoic colorants<br>- the manufacture of dyes, pesticides, and polymer	ECHA (2023), Rivera-Utrilla et al. (2002), Shiyun et al. (2002)
olmesartan	both	- used against hypertension	ECHA (2023), Wishart et al. (2018)
p-cumenesulfonic acid	not specified	- in cleaning and personal care products<br>- in paint and related products	EPA (n.d.), Stanton et al. (2007)
primidone	pharmaceuticals	- antiepileptic agent	Wishart et al. (2018)
2-pyrrolidone	industrial chemicals	- by consumers, in articles, by professional workers, in formulation or re-packing, at industrial sites, and in manufacturing<br>- as solvents or co-solvents in the manufacture of pesticides<br>- in textiles, coatings, electronics, and pharmaceuticals	ECHA (2023), Li et al. (2018)
saccharine	both	- artificial sweetener<br>- by consumers, by professional workers, in formulation or re-packing, at industrial sites, and in manufacturing	ECHA (2023), Wishart et al. (2018)
sulfamethoxazole	pharmaceuticals	- against multiple infections, e.g. pneumocystis	WHO (n.d.), Wishart et al. (2018)
tetrafluoroborate	not specified	- in ionic liquids	Freire et al. (2010)

theophylline	both	- against asthma, chronic obstructive pulmonary disease (COPD), or similar	ECHA (2023), Wishart et al. (2018)
trifluoroacetic acid	industrial chemicals	- by professional workers, in formulation or re-packing, at industrial sites, and in manufacturing<br>- for peptide synthesis as ion-pairing agent<br>- for purification of pharmaceutical and biotechnological products	ECHA (2023), Kaiser and Rohrer (2004), Tipps et al. (2012)
tris(2-chloroethyl) phosphate	industrial chemicals	- in articles, by professional workers, and at industrial sites<br>- flame retardant, plasticizer, and viscosity regulator	ECHA (2023), European Commission (2012)
tris(1-chloromethylethyl) phosphate	industrial chemicals	- by consumers, in articles, by professional workers, in formulation or re-packing, at industrial sites, and in manufacturing<br>- flame retardant	ECHA (2023), Yan et al. (2022)
venlafaxine	both	- antidepressant agent	ECHA (2023), Wishart et al. (2018)
vincubine	industrial chemicals	- at industrial sites, and in manufacturing<br>- for light stabilizers	ECHA (2023), Taylor and Milligan (1989)
3,4-xylenesulfonic acid	not specified	- acid catalyst used in foundries<br>- in cleaning and personal care products (e.g. cosmetic ingredient)	Bergfeld et al. (2011), Stanton et al. (2007), Zhang et al. (2014)

Table S3 Dilution and spike concentrations per sample.

Sample		Dilution in MeOH:MQ (50:50, *v:v*) / spike concentration in µg L^{-1}			
		1:10	1:100	1:1,000	1:10,000
industrial wastewater	traffic related industry #1	yes (10 µg L^{-1})	yes (1 µg L^{-1})	yes (1 µg L^{-1})	
	traffic related industry #2	yes (10 µg L^{-1})	yes (1, 10 µg L^{-1})		
	metal production and finishing	yes (10 µg L^{-1})	yes (1, 10 µg L^{-1})	yes (10 µg L^{-1})	yes (10 µg L^{-1})
	diverse industry	yes (10 µg L^{-1})	yes (1 µg L^{-1})	yes (10 µg L^{-1})	yes (1 µg L^{-1})
	bakery	yes (10 µg L^{-1})	yes (1 µg L^{-1})	yes (10 µg L^{-1})	yes (1 µg L^{-1})
	waste management	yes (10 µg L^{-1})	yes (1 µg L^{-1})		
	cleaning	yes (10 µg L^{-1})	yes (1 µg L^{-1})	yes (10 µg L^{-1})	yes (10 µg L^{-1})
clinical wastewater	hospital #1	yes (10 µg L^{-1})	yes (1 µg L^{-1})		
	hospital #2	yes (10 µg L^{-1})	yes (1 µg L^{-1})	yes (1, 10 µg L^{-1})	
	hospital #3	yes (10 µg L^{-1})	yes (1 µg L^{-1})	yes (1, 10 µg L^{-1})	
	nursing home #1	yes (10 µg L^{-1})	yes (1, 10 µg L^{-1})	yes (10 µg L^{-1})	yes (1 µg L^{-1})
	nursing home #2	yes (10 µg L^{-1})	yes (1 µg L^{-1})	yes (10 µg L^{-1})	
	nursing home #3	yes (10 µg L^{-1})	yes (1 µg L^{-1})	yes (1, 10 µg L^{-1})	
domestic wastewater	households #1	yes (10 µg L^{-1})	yes (1 µg L^{-1})	yes (1 µg L^{-1})	
	households #2	yes (10 µg L^{-1})	yes (1 µg L^{-1})	yes (1 µg L^{-1})	
	households #3	yes (10 µg L^{-1})	yes (1 µg L^{-1})	yes (1 µg L^{-1})	
	households #4	yes (10 µg L^{-1})	yes (1 µg L^{-1})	yes (1, 10 µg L^{-1})	yes (10 µg L^{-1})
	households #5	yes (10 µg L^{-1})	yes (1 µg L^{-1})	yes (1, 10 µg L^{-1})	
	households #6	yes (10 µg L^{-1})	yes (1 µg L^{-1})		

Table S4 SFC-MS parameter of analyzed substances.

Analyte	ESI	t_R in min	Method	Quantifier	Qualifier	Cone voltage in V	Collision energy in eV
acesulfame	-	8.7	DIOL	82	78	8	14 / 22
acetoguanamine	+	5.4	BEH	43	68	30	16 / 16
1-adamantanamine	+	7.2	BEH	135	79	42	24 / 24
3-aminobenzenesulfonic acid	-	8.7	DIOL	108	80	56	18 / 24
1H-benzotriazole	+	4.1	BEH	65	92	2	20 / 14
bistriflimide	-	8.3	DIOL	147	78	8	24 / 36
carbamazepine	+	4.7	BEH	179	165	4	30 / 38
1-cyanoguanidine	+	5.7	BEH	68	-	20	12
cyanuric acid	-	5.0	BEH	42	-	36	14
diatrizoic acid	+	9.5	BEH	361	233	46	16 / 40
1,4-diazabicyclo[2.2.2] octane	+	3.8	BEH	70	56	34 / 8	14 / 31
dibutylphosphate	-	7.2	DIOL	153	79	26	14 / 26
diclofenac	-	4.1	DIOL	250	214	16	20 / 20
ε-caprolactam	+	3.8	BEH	79	44	32	12 / 16
ethenesulfonic acid	-	8.7	DIOL	80	43	42	14 / 14
guanylurea	+	6.9	BEH	60	86	24	8 / 6
losartan	+	6.4	BEH	207	180	8	24 / 38
melamine	+	6.8	BEH	85	43	2	16 / 20
metformin	+	9.8	BEH	60	71	6	12 / 18
4-methylbenzenesulfonic acid	-	7.2	BEH	107	80	44	18 / 26
methyl hydrogen sulfate	-	8.8	DIOL	80	96	46	16 / 16

1,5-naphthalene disulfonic acid	-	10.7	BEH	207	143	2	24 / 30
naphthalene-1-sulfonic acid	-	7.5	BEH	143	80	36	20 / 28
olmesartan	+	9.7	BEH	207	235	4	26 / 20
p-cumenesulfonic acid	-	8.2	DIOL	184	119	10	16 / 36
primidone	+	5.3	BEH	162	91	4	10 / 24
2-pyrrolidone	+	4.0	BEH	44	69	18	14 / 10
saccharine	-	9.1	DIOL	106	42	52	16 / 18
sulfamethoxazole	+	6.0	DIOL	92	156	12 / 16	37 / 21
tetrafluoroborate	-	8.6	DIOL	87	86	2	54 /54
theophylline	+	4.4	BEH	124	96	40	18 / 14
trifluoroacetic acid	-	7.9	DIOL	69	-	2	22
tris(2-chloroethyl) phosphate	+	3.0	BEH	99	223	34	20 / 12
tris(1-chloromethyl ethyl) phosphate	+	2.8	BEH	99	175	6	18 / 12
venlafaxine	+	7.0	BEH	58	121	32	18 / 26
vincubine	+	3.8	BEH	58	83	2	12 / 16
3,4-xylenesulfonic acid	-	8.4	DIOL	80	121	50	24 / 20
2-acrylamido-2-methyl-propanesulfonic acid	-	3.9	DIOL	205	249	18	24 / 24
3-(acryloyloxy)-1-propanesulfonic acid	-	8.5	DIOL	95	80	46	18 / 24
3-(allyloxy)-2-hydroxy-1-propanesulfonic acid	-	8.7	DIOL	135	80	14	18 / 26
ametryn	+	2.9	BEH	96	68	12	24 / 36
4-amino-3-methylbenzenesulfonic acid	-	8.4	BEH	186	80	64	20 / 24
3,5-bis(methoxycarbonyl) benzenesulfonic acid	-	8.7	DIOL	278	209	4	24 / 32
climbazole	+	3.7	BEH	69	197	16	18 / 14
dicyclohexyl sulfosuccinate	-	6.9	BEH	81	-	34	24
2-[2-(dimethylamino) ethoxy]ethanol	+	8.3	BEH	72	42	6 / 8	14 / 49
1,3-di-o-tolylguanidine	+	6.9	BEH	133	108	44	22 / 22
1,3-diphenylguanidine	+	7.1	BEH	119	77	2	22 / 36
3,5-ditertbutyl salicylic acid	-	8.5	DIOL	150	209	66	26 / 22
1-ethylpyridinium	+	12.5	BEH	80	53	10	14 / 20
ethyltrimethylammonia	+	12.7	BEH	58	73	28	16 / 12
gabapentin lactam	+	3.8	BEH	95	67	10	20 / 26
hexafluorophosphate	-	8.2	DIOL	71	80	18	10 / 38
2-[4-(2-hydroxyethyl)-1-piperazine]ethanesufonic acid	+	11.0	BEH	131	88	10	18 / 22
3-(methacryloylamino)-N,N,N-trimethyl-1-propanaminium chloride	+	12.7	BEH	126	-	2	20
4,4'-methylenedianiline	+	4.6	DIOL	106	89	20	24 / 24
1-methyl-1-octylpyrrolidinium	+	10.5	BEH	86	57	42	20 / 24
2-methyl-2-propene-1-sulfonic acid	-	9.2	DIOL	145	144	10	6 / 6
N-[3-(dimethylamino) propyl]-2-methylacryl amide	+	7.9	BEH	69	126	2	20 / 24

N,N-dibutyl-N-methyl-1-butanaminium	+	8.9	BEH	100	58	92	22 / 30
N,N,N-trimethyl(phenyl)methaneaminium	+	11.6	BEH	91	58	2	16 / 12
oxipurinol	+	7.2	BEH	136	80	2	14 / 24
perfluoropropionic acid	-	7.3	DIOL	169	213	2	12 / 12
perfluorobutanoic acid	-	10.6	BEH	207	143	2	24 / 30
1-propylpyridinium	+	11.9	BEH	80	43	14	14 / 14
tricyanomethanide	-	7.8	BEH	64	-	44	18
tris(trifluoromethylsulfonyl)methanide	-	7.7	DIOL	119	163	2	12 / 12

Table S5 Eluent gradient of both used methods (left: BEH column, right: Torus DIOL column).

Total Time (min)	A (%)	B (%)
0.0	99.0	1.0
1.0	99.0	1.0
12.0	50.0	50.0
14.5	50.0	50.0
14.7	99.0	1.0
17.2	99.0	1.0

Total Time (min)	A (%)	B (%)
0.0	98.0	2.0
1.0	98.0	2.0
8.0	70.0	30.0
9.0	70.0	30.0
9.1	98.0	2.0
10.0	98.0	2.0

Method validation

For method validation (Table S6) the following parameters were considered. Linear range and the coefficient of determination (R^2) were derived from a 7-point calibration. For determination of the method detection (LOD) and method quantification limit (LOQ) either instrumental detection/quantification limit (eq 1) or the concentration of the process blank (eq 2) was used. Matrix effects were calculated according to eq 3 for all 19 wastewater samples. Therefore, a concentration between 1 and 10 µg L^{-1} of reference standard mix was spiked based on the concentration present in the given sample dilution. To determine the trueness the ratio between measured spiked concentration and the actual spiked concentration was calculated (eq 4). For precision, relative standard deviation (RSD) of a 1 µg L^{-1} reference standard was calculated within a measured sequence. Herein, robustness was defined as RSD from the reference standards measured over a two-month period (eq 5). Values below the instrumental quantification limit were excluded. Furthermore, recovery rates of the reference standards were calculated.

$$\mathrm{LOD/LOQ}\left(\frac{\mu g}{L}\right) = \frac{\frac{3 \text{ or } 9 * c_{\text{reference standard}}}{s/n} * 2}{\mathrm{ME}} * \text{dilution factor} \quad (1)$$

$$\mathrm{LOD/LOQ}\left(\frac{\mu g}{L}\right) = c_{\text{blank}} + 3 \text{ or } 9 * \mathrm{SD}_{\text{blank}} \quad (2)$$

$$\mathrm{ME} = \left(\frac{c_{\text{spiked sample}} - c_{\text{non spiked sample}}}{c_{\text{reference standard}}} * 100\%\right) - 100\% \quad (3)$$

$$\delta = \frac{c_{\text{spiked sample}} - c_{\text{non spiked sample}}}{c_{\text{spiked}}} * \mathrm{ME} \quad (4)$$

$$\mathrm{RSD}\ (\%) = \frac{\mathrm{SD}}{c_{\text{mean}}} * 100 \quad (5)$$

Table S6 Method validation parameters. Detailed information on how the values were determined are shown above.

Analyte	Linear range in µg/L	Coefficient of determination R^2	Method detection limit (LOD) in µg/L	Method quantification limit (LOQ) in µg/L	Matrix effects in % (n = 19)	Trueness (n = 19)	Precision RSD in % (n = 4)[a)]	Robustness RSD in % (n = 7)[a) b)]
ACE	0.1 - 10	0.999	0.25	0.76	2.0 ± 24.9	1.01 ± 0.08	11.7	22.4
AG	0.05 - 10	1.000	0.16	0.47	7.2 ± 5.9	1.01 ± 0.00	3.0	2.0
ATA	0.05 - 10	0.999	0.12	0.36	7.0 ± 3.9	0.98 ± 0.00	4.1	6.4
MA	1 - 10	0.997	1.54	4.62	-1.4 ± 31.5	1.04 ± 0.04	0.1	31.6
BTZ	0.01 - 10	1.000	0.79	2.37	11.9 ± 7.8	1.00 ± 0.02	5.7	5.8
NtF2	0.5 - 10	1.000	0.03	0.08	-3.8 ± 4.0	1.01 ± 0.00	6.5	6.7
CBZ	0.01 - 10	0.999	0.22	0.67	-1.7 ± 6.1	1.02 ± 0.00	1.0	5.0
CG	0.01 - 10	1.000	0.01	0.02	-8.7 ± 15.3	1.02 ± 0.00	8.2	8.3
CA	0.5 - 10	0.990	0.91	2.74	-10.7 ± 18.1	1.05 ± 0.00	28.3	29.7
DZA	0.05 - 10	1.000	0.41	1.24	-6.5 ± 18.2	1.00 ± 0.02	19.1	16.3
DABCO	0.5 - 10	0.998	2.97	8.92	7.2 ± 11.9	0.94 ± 0.12	11.5	12.8
DBP	0.1 - 10	0.998	0.62	1.86	-8.2 ± 7.0	1.02 ± 0.00	28.4	43.5
DCF	0.5 - 10	0.999	5.61	16.84	-3.9 ± 6.2	1.01 ± 0.00	-	23.6
εCL	0.01 - 10	0.999	0.90	2.71	7.5 ± 11.5	0.95 ± 0.11	32.0	25.3
ESA	0.5 - 10	0.997	0.75	2.24	-2.6 ± 8.5	0.97 ± 0.00	41.5	65.1
GUA	0.1 - 10	0.999	3.26	9.79	12.8 ± 15.6	1.03 ± 0.03	5.7	9.8
LOS	0.05 - 10	0.997	0.04	0.12	0.5 ± 4.7	1.04 ± 0.00	2.2	5.6
MEL	0.01 - 10	1.000	0.23	0.70	4.7 ± 6.5	1.00 ± 0.02	4.5	3.4
MET	0.05 - 10	0.998	0.45	1.34	11.5 ± 16.9	1.11 ± 0.23	7.8	9.4
PTSS	0.1 - 10	0.999	2.23	6.69	-51.7 ± 7.9	1.00 ± 0.00	-	-
MHS	0.5 - 10	0.999	16.10	48.30	5.5 ± 34.8	1.00 ± 0.07	-	-
NDSA	1 - 10	1.000	23.30	69.89	-7.0 ± 12.2	1.00 ± 0.00	-	-
NSA	0.5 - 10	0.999	3.67	11.01	-50.7 ± 17.1	0.98 ± 0.00	4.4	11.5
OLM	0.01 - 10	1.000	0.12	0.37	-4.7 ± 6.1	1.01 ± 0.00	8.4	8.8
CSA	1 - 10	1.000	1.66	4.98	-3.5 ± 58.1	1.54 ± 0.54	-	-
PRI	0.01 - 10	1.000	0.54	1.62	2.8 ± 5.1	1.00 ± 0.00	2.8	6.7
2PYR	1 - 10	0.979	5.42	16.27	128 ± 21.2	1.07 ± 0.00	-	-
SAC	0.5 - 10	1.000	0.89	2.66	16.3 ± 66.3	1.02 ± 0.04	34.7	33.3
SMX	0.1 - 10	0.999	0.09	0.27	4.2 ± 6.8	1.00 ± 0.00	10.1	12.0
BF_4	0.5 - 10	1.000	0.43	1.29	-15.7 ± 19.3	0.99 ± 0.02	26.2	25.3
THEO	0.01 - 10	0.999	0.15	0.44	0.4 ± 11.9	1.00 ± 0.00	1.9	7.8
TFA	0.5 - 10	0.990	3.40	10.20	14 ± 11.3	0.94 ± 0.00	16.0	22.8

TCEP	0.1 - 10	0.998	0.49	1.47	2.2 ± 5.0	1.01 ± 0.00	3.0	1.9
TCPP	0.05 - 10	0.998	1.07	3.22	17.7 ± 6.7	0.97 ± 0.00	-	-
VEN	0.01 - 10	1.000	0.08	0.25	14.4 ± 6.0	1.01 ± 0.00	7.1	6.0
VIN	1 - 10	0.999	0.11	0.34	-0.4 ± 7.3	0.95 ± 0.00	5.9	14.9
XSA	0.5 - 10	0.994	0.74	2.21	9.1 ± 19.4	1.06 ± 0.25	-	-
AAMPS	0.1 - 10	0.999	0.16	0.47	-2.9 ± 10.9	1.01 ± 0.00	47.1	39.1
AOPSO3	0.1 - 10	1.000	0.11	0.33	-13.4 ± 7.7	1.01 ± 0.00	44.9	40.0
AHP	0.5 - 10	0.999	1.08	3.25	-5.6 ± 8.8	0.98 ± 0.00	30.4	50.9
ATY	0.01 - 10	1.000	0.11	0.32	1.4 ± 4.1	0.99 ± 0.00	2.8	2.8
AMB	0.1 - 10	0.996	10.97	32.91	-9.7 ± 11.4	1.05 ± 0.00	-	-
SIP	0.1 - 10	0.998	0.07	0.20	-2.1 ± 8.7	0.99 ± 0.00	32.5	26.3
CLIM	0.01 - 10	1.000	0.62	1.85	1.8 ± 7.0	0.99 ± 0.00	4.7	4.8
DCHSS	1 - 10	0.993	0.49	1.46	-18.1 ± 7.9	0.96 ± 0.00	21.0	30.9
DMAEE	0.05 - 10	0.999	0.01	0.00	36.1 ± 4.5	1.01 ± 0.00	4.0	4.1
DIOTOG	0.05 - 10	0.997	0.18	0.55	38.3 ± 8.2	0.97 ± 0.00	24.7	29.5
DPG	0.01 - 10	0.999	1.96	5.89	22.9 ± 8.6	1.01 ± 0.00	5.6	16.3
DTBSA	0.5 - 10	0.992	0.95	2.85	-5.7 ± 7.9	0.95 ± 0.00	9.2	7.1
PYC2	0.5 - 10	0.996	0.64	1.92	197.9 ± 10.4	1.04 ± 0.00	5.7	9.8
AC5	0.5 - 10	0.996	2.60	7.79	176.6 ± 9.7	1.03 ± 0.00	-	-
GALA	0.01 - 10	1.000	0.16	0.48	10.0 ± 4.7	1.01 ± 0.00	1.9	3.8
PF6	0.1 - 10	0.999	0.46	1.39	3.2 ± 3.8	1.00 ± 0.00	-	-
HEPES	0.05 - 10	1.000	2.31	6.94	7.5 ± 5.4	0.99 ± 0.00	7.1	6.8
MAPTAC	0.05 - 10	0.999	0.53	1.59	61.1 ± 5.2	1.03 ± 0.00	2.1	3.2
MDA	0.1 - 10	0.998	1.61	4.82	-1.8 ± 6.9	1.00 ± 0.00	5.3	7.7
PLC9	0.05 - 10	0.999	7.61	22.83	25.4 ± 7.2	1.02 ± 0.00	-	-
MPSA	0.1 - 10	0.999	0.12	0.35	-13.6 ± 6.3	1.00 ± 0.00	44.2	41.2
MAPMA	0.05 - 10	0.999	1.08	3.25	15.8 ± 7.5	1.03 ± 0.00	3.2	6.7
AC13	1 - 10	0.991	1.83	5.50	31.5 ± 14.2	0.95 ± 0.00	3.1	5.2
BETMAC	0.05 - 10	0.999	0.02	0.06	28.4 ± 4.8	1.02 ± 0.00	4.1	6.3
OXI	0.05 - 10	0.999	0.15	0.45	11.6 ± 3.2	0.98 ± 0.00	6.5	7.8
PFBA	0.5 - 10	0.999	5.04	15.11	-37.2 ± 8.6	1.01 ± 0.00	-	-
PFPrA	0.5 - 10	1.000	0.55	1.65	7.5 ± 4.6	1.00 ± 0.00	5.7	12.5
PYC3	0.5 - 10	0.997	0.16	0.47	91.5 ± 6.7	1.03 ± 0.00	3.2	3.2
TCM	1 - 10	0.994	4.61	13.83	-12.3 ± 14.3	0.96 ± 0.00	-	-
TFSM	0.1 - 10	0.998	0.09	0.27	3.2 ± 7.9	0.98 ± 0.00	2.1	3.1

[a] Values below the instrumental quantification limit were excluded.

[b] over 2-month period

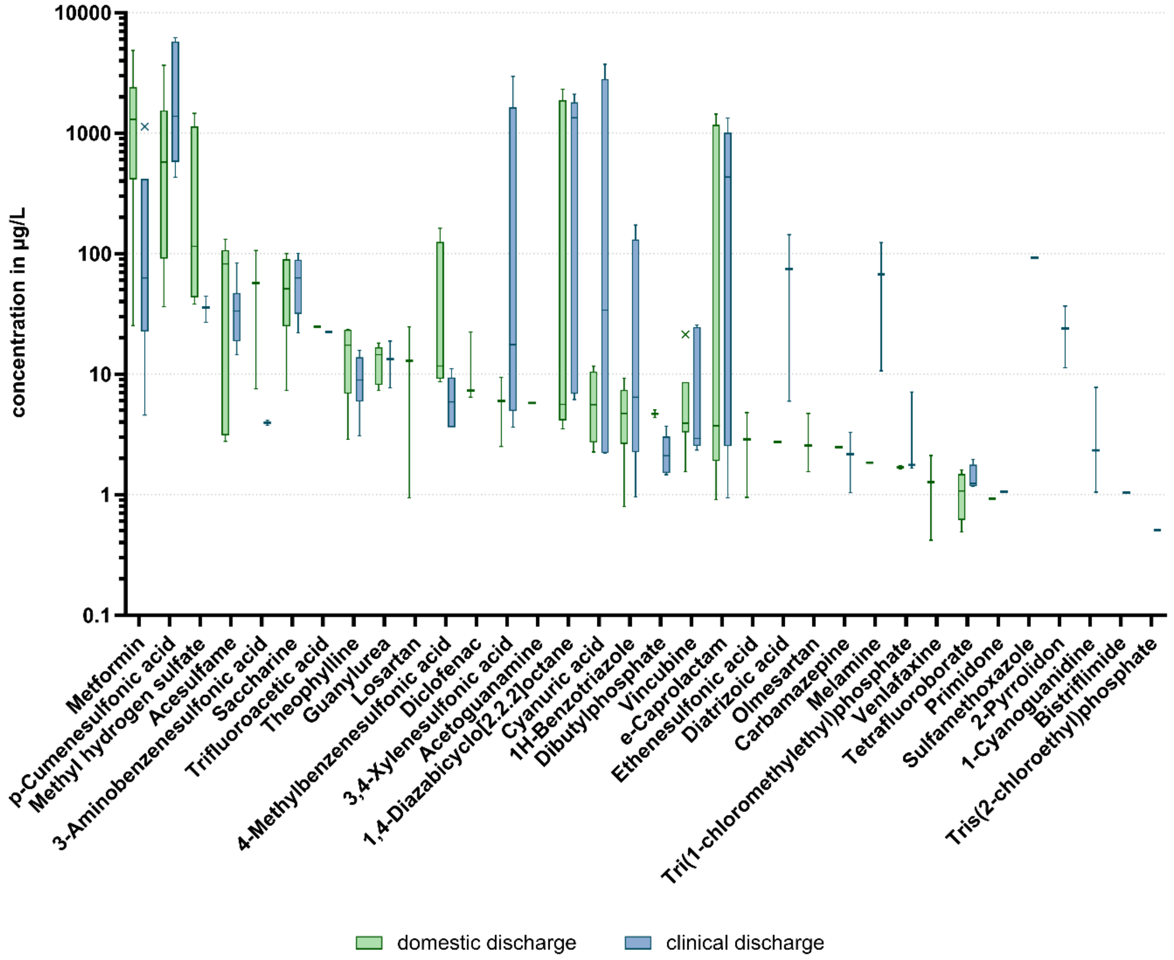

Fig. S1 Concentrations of PM chemicals in domestic (green) and clinical (blue) discharge.

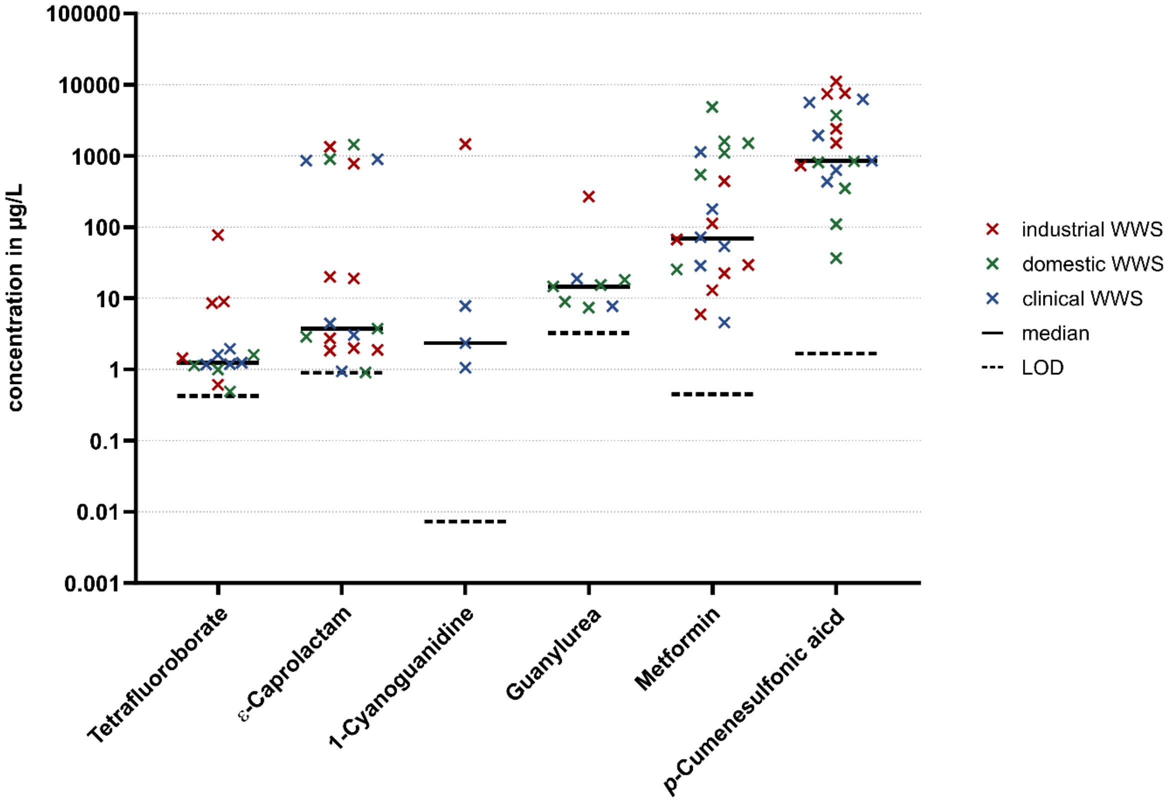

Fig. S2 Concentrations of selected analytes. Tetrafluoroborate (BF_4), ε-caprolactam (εCL), cyanoguanidine (CG), guanylurea (GUA), metformin (MET), and *p*-cumenesulfonic acid (CSA) in industrial (red), domestic (green) and clinical (blue) wastewater samples (WWS). LOD represented by dashed line.

References

Amer, I., Mokrani, T., Jewell, L., Young, D.A., Vosloo, H.C.M., 2016. Oxidative copolymerization of p-phenylenediamine and 3-aminobenzenesulfonic acid. *Tetrahedron Letters* **57**, 426–430. https://doi.org/10.1016/j.tetlet.2015.12.056

Basavaiah, D., Dharma Rao, P., Suguna Hyma, R., 1996. The Baylis-Hillman reaction: A novel carbon-carbon bond forming reaction. *Tetrahedron* **52**, 8001–8062. https://doi.org/10.1016/0040-4020(96)00154-8

Baylis, A.B., Hillman, M.E.D., 1972. Process for producing acrylic compounds (in German). German Patent 2155113.

Bergfeld, W.F., Belsito, D.V., Klaassen, C.D., Hill, R., Liebler, D., Marks, J.G., Shank, R.C., Slaga, T.J., Snyder, P.W., Andersen, F.A., 2011. Safety Assessment of Xylene Sulfonic Acid, Toluene Sulfonic Acid, and Alkyl Aryl Sulfonate Hydrotropes as Used in Cosmetics. *Int J Toxicol* **30**, 270S-283S. https://doi.org/10.1177/1091581811429085

Braekevelt, E., Lau, B.P.-Y., Feng, S., Ménard, C., Tittlemier, S.A., 2011. Determination of melamine, ammeline, ammelide and cyanuric acid in infant formula purchased in Canada by liquid chromatography-tandem mass spectrometry. *Food Additives & Contaminants: Part A* **28**, 698–704. https://doi.org/10.1080/19440049.2010.545442

Chellquist, E.M., Nelson, W.O., Storflor, H.L., 1997. Assay and purity analysis of diatrizoate sodium in drug product by LC. *Journal of Pharmaceutical and Biomedical Analysis* **16**, 39–45. https://doi.org/10.1016/S0731-7085(97)00007-1

Cornet, S.M., May, I., Redmond, M.P., Selvage, A.J., Sharrad, C.A., Rosnel, O., 2009. Coordination of bistriflimide and triflate to the uranyl bis(diphenylphosphino)methane dication, {UO2(DPPMO2)2}2+. *Polyhedron* **28**, 363–369. https://doi.org/10.1016/j.poly.2008.10.070

Davis, L.N., Santodonato, J., Howard, P.H., Saxena, J., 1977. Investigation of Selected Potential Environmental Contaminents: Benzotriazole. U.S. Environmental Protection Agency (ed.). EPA 560/2-77-001, https://nepis.epa.gov/Exe/ZyPDF.cgi/910127PS.PDF?Dockey=910127PS.PDF, retrieved December 12, 2023

European Chemicals Agency (ECHA), 2023. Substance Infocard. https://echa.europa.eu/de/home, retrieved September 22, 2023.

European Commission, Directorate-General for Health and Consumers, 2012. Opinion on tris(2-chloroethyl)phosphate (TCEP) in toys. https://data.europa.eu/doi/10.2772/44937, retrieved December 12, 2023.

Fang, Z., Zheng, X., Li, L., Qi, J., Wu, W., Lu, Y., 2022. Ionic Liquids: Emerging Antimicrobial Agents. *Pharm Res* **39**, 2391–2404. https://doi.org/10.1007/s11095-022-03336-5

Freire, M.G., Neves, C.M.S.S., Marrucho, I.M., Coutinho, J.A.P., Fernandes, A.M., 2010. Hydrolysis of Tetrafluoroborate and Hexafluorophosphate Counter Ions in Imidazolium-Based Ionic Liquids. *J. Phys. Chem. A* **114**, 3744–3749. https://doi.org/10.1021/jp903292n

Kaiser, E., Rohrer, J., 2004. Determination of residual trifluoroacetate in protein purification buffers and peptide preparations by ion chromatography. *Journal of Chromatography A* **1039**, 113–117. https://doi.org/10.1016/j.chroma.2004.03.044

Kasetti, Y., Bharatam, P.V., 2013. Pharmacophoric features of drugs with guanylurea moiety: an electronic structure analysis. *J Mol Model* **19**, 1865–1874. https://doi.org/10.1007/s00894-012-1743-2

Li, H., Jiang, Z., Cao, X., Su, H., Shao, H., Jin, F., Zheng, L., Abd El-Aty, A.M., Wang, J., 2018. SPE/GC–MS Determination of 2-Pyrrolidone, N-Methyl-2-pyrrolidone, and N-Ethyl-2-pyrrolidone in Liquid Pesticide Formulations. *Chromatographia* **81**, 359–364. https://doi.org/10.1007/s10337-017-3435-7

Molz, T., Rehm, H.-J., Vogt, T., Geke, J., 1996. Process for denaturing and coagulating paints. U.S. Patent 5547587.

Oberley, W.J., 1983. Non-resinous, uncured tire retardant and products produced therewith. 4,373,010.

Rivera-Utrilla, J., Sánchez-Polo, M., Zaror, C.A., 2002. Degradation of naphthalenesulfonic acids by oxidation with ozone in aqueous phase. *Phys. Chem. Chem. Phys.* **4**, 1129–1134. https://doi.org/10.1039/b108194b

Schulze, S., Zahn, D., Montes, R., Rodil, R., Quintana, J.B., Knepper, T.P., Reemtsma, T., Berger, U., 2019. Occurrence of emerging persistent and mobile organic contaminants in European water samples. *Water Research* **153**, 80–90. https://doi.org/10.1016/j.watres.2019.01.008

Sepehrianazar, A., Güven, O., 2023. Synthesis and characterization of poly(vinyl sulfonic acid) in different pH values. *Polym. Bull.* **80**, 3005–3020. https://doi.org/10.1007/s00289-022-04190-6

Shiyun, Z., Xuesong, Z., Daotang, L., 2002. Ozonation of naphthalene sulfonic acids in aqueous solutions. Part I: elimination of COD, TOC and increase of their biodegradability. *Water Research* **36**, 1237–1243. https://doi.org/10.1016/S0043-1354(01)00331-1

van Soestbergen, M., Baukh, V., Erich, S.J.F., Huinink, H.P., Adan, O.C.G., 2014. Release of cerium dibutylphosphate corrosion inhibitors from highly filled epoxy coating systems. *Progress in Organic Coatings* **77**, 1562–1568. https://doi.org/10.1016/j.porgcoat.2013.12.018

Stanton, K., Tibazarwa, C., Certa, H., Greggs, W., Hillebold, D., Jovanovich, L., Woltering, D., Sedlak, R., 2009. Environmental Risk Assessment of Hydrotropes in the United States, Europe and Australia. *Integr Environ Assess Manag* **6**, 155–163. https://doi.org/10.1897/IEAM_2009-019.1

Taylor, D.B., Milligan, B., 1989. Process for preparing triacetone amine and other oxopiperidines. U.S. Patent 4831146.

Tipps, M.E., Iyer, S.V., John Mihic, S., 2012. Trifluoroacetate is an allosteric modulator with selective actions at the glycine receptor. *Neuropharmacology* **63**, 368–373. https://doi.org/10.1016/j.neuropharm.2012.04.011

Turk, S.C.H.J., Kloosterman, W.P., Ninaber, D.K., Kolen, K.P.A.M., Knutova, J., Suir, E., Schürmann, M., Raemakers-Franken, P.C., Müller, M., de Wildeman, S.M.A., Raamsdonk, L.M., van der Pol, R., Wu, L., Temudo, M.F., van der Hoeven, R.A.M., Akeroyd, M., van der Stoel, R.E., Noorman, H.J., Bovenberg, R.A.L., Trefzer, A.C., 2016. Metabolic Engineering toward Sustainable Production of Nylon-6. *ACS Synth. Biol.* **5**, 65–73. https://doi.org/10.1021/acssynbio.5b00129

U.S. Environmental Protection Agency (EPA), n.d. Comptox Chemicals Dashboard. https://comptox.epa.gov/dashboard/, retrieved October 11, 2023.

Wishart, D.S., Feunang, Y.D., Guo, A.C., Lo, E.J., Marcu, A., Grant, J.R., Sajed, T., Johnson, D., Li, C., Sayeeda, Z., Assempour, N., Iynkkaran, I., Liu, Y., Maciejewski, A., Gale, N., Wilson, A., Chin, L., Cummings, R., Le, D., Pon, A., Knox, C., Wilson, M., 2018. DrugBank 5.0: a major update to the DrugBank database for 2018. *Nucleic Acids Research* **46**, D1074–D1082. https://doi.org/10.1093/nar/gkx1037

World Health Organization (WHO), n.d. Model List of Essential Medicines. https://list.essentialmeds.org/, retrieved October 11, 2023.

Yan, J., Zhao, Z., Xia, M., Chen, S., Wan, X., He, A., Daniel Sheng, G., Wang, X., Qian, Q., Wang, H., 2022. Induction of lipid metabolism dysfunction, oxidative stress and inflammation response by tris(1-chloro-2-propyl)phosphate in larval/adult zebrafish. *Environment International* **160**, 107081. https://doi.org/10.1016/j.envint.2022.107081

Zhang, H., Zhao, H., Zheng, K., Li, X., Liu, G., Wang, Y., 2014. Diminishing hazardous air pollutant emissions from pyrolysis of furan no-bake binders using methanesulfonic acid as the binder catalyst. *J Therm Anal Calorim* **116**, 373–381. https://doi.org/10.1007/s10973-013-3553-x

Zheng, Y., Zhou, X., Luo, Y., Yu, P., 2020. Electrodeposition of nickel in air- and water-stable 1-butyl-3-methylimidazolium dibutylphosphate ionic liquid. *RSC Adv.* **10**, 16576–16583. https://doi.org/10.1039/D0RA00351D

Zhu, H., Kannan, K., 2020. Occurrence and distribution of melamine and its derivatives in surface water, drinking water, precipitation, wastewater, and swimming pool water. *Environmental Pollution* **258**, 113743. https://doi.org/10.1016/j.envpol.2019.113743
